# Exploring the Emerging Role of the Gut Microbiota and Tumor Microenvironment in Cancer Immunotherapy

**DOI:** 10.3389/fimmu.2020.612202

**Published:** 2021-01-07

**Authors:** Qin Qiu, Yuqi Lin, Yucui Ma, Xiaoling Li, Juan Liang, Zhiyan Chen, Kaifeng Liu, Yuge Huang, Hui Luo, Riming Huang, Lianxiang Luo

**Affiliations:** ^1^ Graduate School, Guangdong Medical University, Zhanjiang, China; ^2^ Guangdong Provincial Key Laboratory of Food Quality and Safety, College of Food Science, South China Agricultural University, Guangzhou, China; ^3^ The Marine Biomedical Research Institute, Guangdong Medical University, Zhanjiang, China; ^4^ Animal Experiment Center, Guangdong Medical University, Zhanjiang, China; ^5^ The First Clinical College, Guangdong Medical University, Zhanjiang, China; ^6^ Department of Pediatrics, The Affiliated Hospital of Guangdong Medical University, Zhanjiang, China; ^7^ The Marine Biomedical Research Institute of Guangdong Zhanjiang, Zhanjiang, China; ^8^ Southern Marine Science and Engineering Guangdong Laboratory (Zhanjiang), Zhanjiang, China

**Keywords:** tumor microenvironment, gut microbiota, immunotherapy, host immunity, programmed cell death protein 1/programmed cell death 1 ligand 1

## Abstract

The tumor microenvironment (TME) is a complex ecosystem, which includes many different types of cells, abnormal vascular systems, and immunosuppressive cytokines. TME serves an important function in tumor tolerance and escapes from immune surveillance leading to tumor progression. Indeed, there is increasing evidence that gut microbiome is associated with cancer in a variety of ways, as specific microbial signatures are known to promote cancer development and influence safety, tolerability, and efficacy of therapies. Studies over the past five years have shown that the composition of the intestinal microbiota has a significant impact on the efficacy of anticancer immunosurveillance, which contribute to the therapeutic activity of cancer immunotherapies based on targeting cytotoxic T lymphocyte protein 4 (CTLA-4) or programmed cell death protein 1 (PD-1)–programmed cell death 1 ligand 1 (PD-L1) axis. In this review, we mainly discuss the impact of TME on cancer and immunotherapy through immune-related mechanisms. We subsequently discuss the influence of gut microbiota and its metabolites on the host immune system and the formation of TME. In addition, this review also summarizes the latest research on the role of gut microbiota in cancer immunotherapy.

## Introduction

Cancer is a major public health problem with high rates of incidence and mortality (1). Over the past few decades, significant progress has been achieved in the field of cancer treatment, the main treatment methods included surgery, chemotherapy, radiotherapy, target therapy, and immunotherapy, among them, immunotherapy has become a research hotspot in recent years among these (2, 3). However, the effect of tumor immunotherapy is largely affected by tumor microenvironment (TME) (4, 5). The TME consisted of a variety of different cell types, which plays an important role in tumor tolerance and the evasion of immune surveillance (6). Studies have shown that multiple cells in TME play a significant role in tumor immunotherapy, including T cell, fibroblasts, natural killer (NK) cell, dendritic cells (DCs), and so on (7–9), NK cells stimulate cDC1 to enter into the TME and promote tumor immune control (10). More than 1,000 microorganisms are living in the human gut, called the gut microbiota, which is closely related to a variety of diseases, for example, chronic inflammation, autoimmunity, cancer, and so on (7, 11). Gut microbiota can shape TME by regulating the immune and hormonal factors of the whole host (7), in other words, host gut microbiota is emerging as a critical modulator of the TME. It is also reported that regulating gut microbiota can enhance the effect of cancer immunotherapy (12). It is worth mentioning that the metabolites of gut microbiota [such as short-chain fatty acids (SCFAs), lipopolysaccharide, and Gallic acid] also have effects on TME and tumor immunosuppressive therapy (13–15). Besides, gut microbiota acts tumor immunotherapy directly or indirectly through immune-related mechanisms. The gut microbiota has been found to inhibit the cancer-suppressing effect of p53 mutations, while antibiotic treatment (elimination of gut microbiota) can restore it (16). Exploring roles of gut microbiota and TME in cancer immunotherapy as well as their interaction based on literature survey, which obtains answer of problems existed in cancer immunotherapy, and clarifies the future research direction of tumor immunotherapy. Meanwhile, it provides a theoretical basis for immunotherapy based on TME and gut microbiota.

## The Role of TME in Cancer Immunotherapy

**Figure 1:**
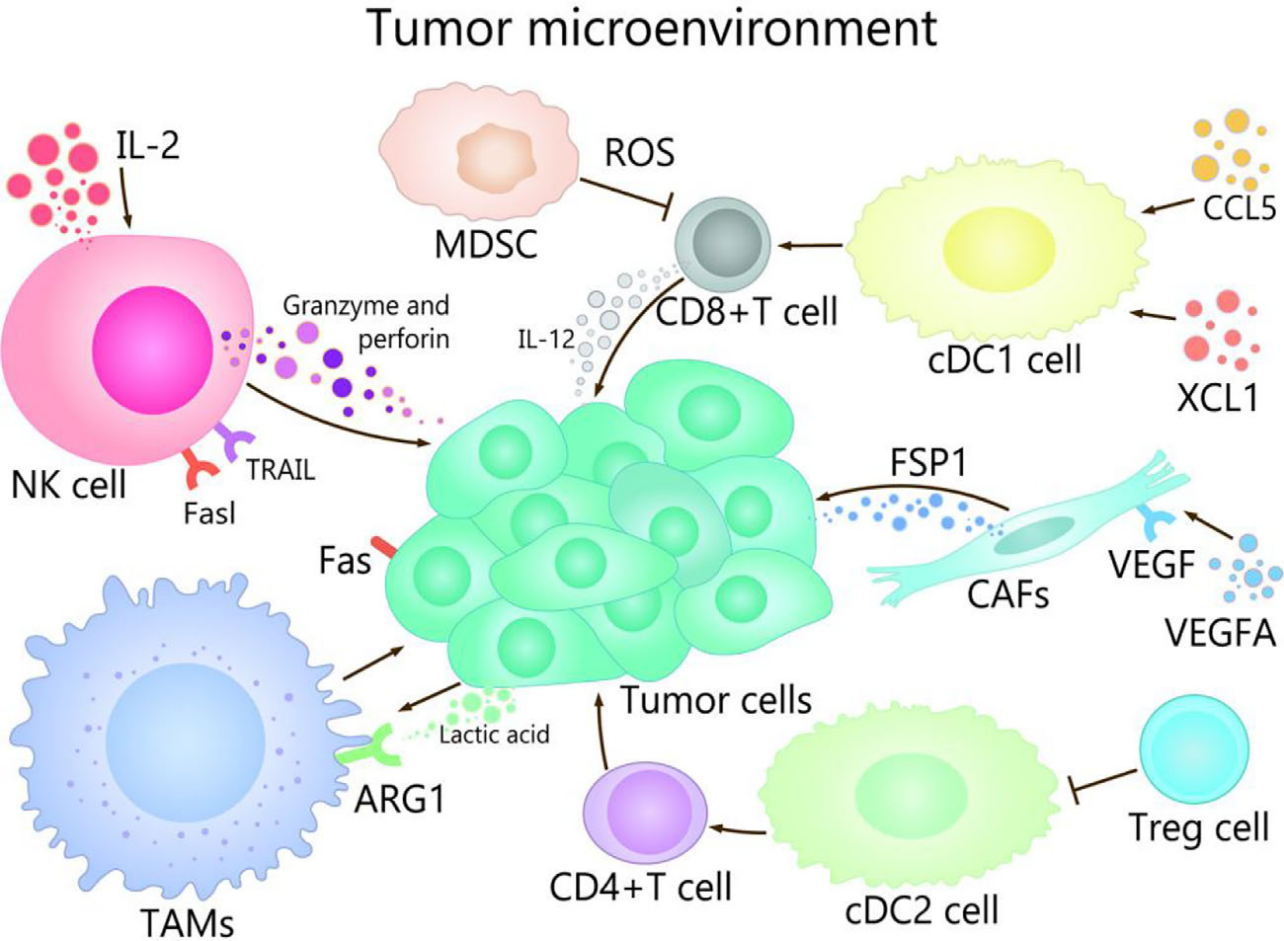
The role of TME in cancer and its immunotherapy. The main cells of the TME in cancer immunity are NK cells, DC cells, CD8 + T cells, Treg cells, fibroblasts, TAMs, and MDSCs. Among them, NK cells induce the death of tumor cells by the ways of releasing perforin and granzyme, secreting tumor necrosis factor-a, and mediating cytotoxicity by TRAIL and Fasl receptors. CDC1 cells are able to promote the differentiation and maturation of CD8^+^ T cells, and cDC1 cells can recruit CCL5 and XCL1, which induce the accumulation of cDC1 cells in the TME, thereby improving the immune control of tumors. IL-2 contributes to enhancing the antitumor activity of NK cells. When CD4 + T cells migrate to lymph nodes, cDC2 can activate CD4^+^ T cell responses. cDC2 resistant CD4 ^+^ T cells can be inhibited by Treg cells. VEGFA activates fibroblasts, which secrete FSP1. TAMs can promote the growth and metastasis of tumor cells through multiple pathways, lactate produced by cancer and acidification of the microenvironment increase ARG1 expression in TAMs. MDSCs affect the ability to respond to non-specific stimulation by producing ROS, etc., which leads to the inability of CD8^+^ T cells combined with pMHC.

TME is composed of immune cells, such as T cells, B cells, and NK cells, a variety of myeloid cell populations including granulocytes, monocytes, macrophages, and DCs, abnormal vasculature and immunosuppressive cytokines, which play different roles in TME (17, 18). The progress of cancer growth, invasion, metastasis, drug resistance, and immune escape are affected by TME (19, 20). For example, TME promotes the occurrence of hepatocellular carcinoma (HCC) in many ways, mainly in that NK cells and DCs participate in immune escape mechanisms, macrophages are involved in promotion of angiogenesis and tissue remodeling, and the production of cytokines and chemokines lead to persistent inflammation-related damage (21–23). On the other hand, well expression of co-inhibitory molecules, especially CTLA-4, PD-1 and PD-L1 are associated with immune-system exhaustion and immune tolerance (24, 25). The regulation of the TME could be used as an effective strategy to prevent as well as treat cancer (2). The purpose of tumor immunotherapy is to stimulate tumor-specific cytotoxic T lymphocytes (CTLs) and subsequent transports, to enable them to reach and persist in TME in order to identify and eliminate malignant target cells (26). TME show different important effects and mechanisms in the tumor and its immunotherapy with different channels (**Figure 1**).

### Dendritic Cells and T Cells

T cells play an irreplaceable role in tumor immunotherapy. T cells through the T cell receptor (TCR) to identify major histocompatibility complex class I/II (MHC- I/MHC-II) molecules that existed on the surface of the specific antigen peptide of malignant cells, which plays an important role in defense against cancer. Tumor targets are mainly achieved by releasing the content of cytolytic granules containing perforin and granzyme. To destroy their targets, CTLs must first migrate to tumor sites, infiltrate tumor tissue, interact with cancer cells, and eventually eliminate transformed cells due to trigger effector functions (27). It is indicated that a better prognosis of lung, melanoma, brain, breast, colorectal cancer is associated with that infiltration of T cells, particularly CD8+ T cells infiltrate into the TME (28, 29). CD8+ T cell mediated anti-tumor immunity of promising cancer immunotherapy, including DC cancer vaccines, adaptability of reactive T cell tumor cell metastasis (ACT) and free of disease checkpoint blockade, such as resistance to PD-1, PD-L1, and resisting CTLA-4 (26), PD-1 therapy is associated with the abundance of CD8+ T cells that support tumor invasion, tumor mutation load, and interferon signaling (30).

Although tumor cells have directly present tumor antigens to MHC-I playing a strong part in the effector function of CD8^+^ T cells, it is necessary that primary CD8^+^ T cells need to be cross-presented by specialized antigen-presenting cells (especially DCs) to maintain the cytotoxic immune response. TME plays a key role not only in initiating the primary immune response, but also in initiating the acquired immune response (31, 32). In fact, DCs in tumors mainly act on T cells and can be divided into two subgroups: conventional DC type 1 (cDC1) and conventional DC type 2 (cDC2) (33). On the one hand, cDC1 can attract T cells in the tumor, to stimulate and magnified the role of tumor specific CD8+ T cells, meanwhile, which can induce the death of tumor cells and the drainage of tumor antigens to lymph nodes (34, 35), where the formation is responsible for the staggered start anti-tumor key DC subtype of CD8+ T cells, so as to achieve the effect of the removal of the tumor (36). On the other hand, conventional type 1 DCs in TME can be induced to support T cell effector function by secreting interleukin-12 (37) and recruiting chemokines such as CCL5 and XCL1 (38, 39).

The response will be activated by cDC2 when CD4^+^ T cells migrate to lymph nodes. Interestingly, regulatory T cells (Tregs) inhibit tumor-responsive activated CD4^+^ T cells by inhibiting cDC2, and Tregs also inhibit mature DCs and prevent their migration to draining lymph nodes (40, 41). The DCs, the main companions of T cells, not only are crucial for initiating a primary immune response, but also play a vital role in initiating acquired immune response in TME (31, 32). Many researches provide new evidence for the wider role of DCs in tumors, including maintaining and supporting effect function in T-cell responses (30). DCs and T cells complement each other in the role of cancer immunotherapy and play an anti-tumor role together. But many unknown mechanisms remain to be further studied in order to better grasp prospects in cancer therapy.

### NK Cell

Although current tumor immunotherapy mainly focuses on T cells, NK cells also are gradually being considered as the key target of tumor immunotherapy. NK cell-based therapy is becoming a safe and effective treatment for some cancers (42). The enhanced anti-tumor response mediated by CD8^+^ T cells was associated with altered the acquired immune response, which related to the interaction between NK cells and immune cells (43, 44). In addition, NK cells recognize and mediate direct cytotoxic activity against tumor cells in early cancer (45). NK cells distinguish tumor cells and viral infections of cancer cells by encoding a series of receptor activation and inhibition of receptors (10). The activated receptor and inhibitory receptor signal are balanced to positively promote the activation of NK cells, which induce the target cell death through releasing granzyme and perforin by exocytosis, the cytotoxicity mediated receptor of by TNF-related apoptosis inducing ligand (TRAIL) and Fas ligand (FasL) (46).

PD-1 is mainly expressed on activated T cells, but also on NK cells, called “PD-1^+^NK cells” accounting for 25% of NK cells, whose expression is increased in a variety of cancers indicating a poor prognosis (47, 48). Blocking the PD-1/PD-L1 interaction with anti–PD-L1 or PD-L1 antibody restores, could reverse the dysfunctional status of PD-1^+^ NK cells and restore the anti-tumor response of NK cells (49, 50). At the same time, the increase in the frequency and activation of NK cells can enhance the response to anti-PD-1 therapy, and the rescue of NK cell activity can enhance the anti-tumor activity of adaptive T cells, thus increasing the overall survival rate of patients with multiple types of cancer (44). The anti-tumor activity of NK cells can be enhanced by cytokines, especially IL-2, but with toxic (51). IL-12 has a huge potential for increasing ADCC-mediated NK cell killing activity in solid tumors (52). It has been found that immunomodulatory drugs like lenalidomide can enhance the cytotoxicity mediated by NK cell and ADCC (16). However, in most studies, a small number and impaired function of NK cells isolated from primary tumors were observed, which was mainly due to the accumulation of suppressors in the TME, which inhibited the anti-tumor activity of NK cells and reduced the recruitment and persistence of NK cells in tumor nests (53). The complex interaction of cancer cells and the immune system has great limitations to the immunogenicity of cancer and promotes immunosuppression, which is the key factor affecting the drug resistance and validity of NK cell therapy. Therefore, a deeper understanding of the complex interactions between NK cells and TME in solid tumors will open up new prospects for cancer treatment (54).

### Tumor-Associated Fibroblasts

Fibroblasts are repeatedly activated by a diverse set of factors secreted from cancer or immune cells, resulting in phenotypic transformation and becoming tumor-associated fibroblasts (CAFs), which are not only the source of immunosuppressive molecules, but also a physical barrier (55). CAFs are indispensable in the immunosuppression within TME and has the role of promoting cancer, thus it has become a target to enhance cancer immunotherapy (56). The occurrence and development of tumors could be inhibited by antifibrotic drugs (57).

CAFs inhibit the activity of CTLs and recruit lymphocytes that produce inflammatory signals to promote cancer progression (58). CAFs can direct or coordinate the infiltration of immune cells directly or through secreted cytokines and surface proteins, or indirectly and coordinate the infiltration of immune cells by depositing various ECM substrates and remodeling matrices, thereby promote cancer (59, 60). For example, the protein-1 (FSP1) secreted by fibroblasts, which cause metastasis of colon and breast cancer, and the factor A (VEGFA) induce the development of cancer cell (61). In addition, CAFs promotes resistance to anticancer drugs or therapies and provides protective or proliferative factors in cancer cells (62). Genetic variation has been found in cancer-associated fibroblasts, which is more genetically stable than tumor cells, making it an alternative target for immunotherapy (63). CAFs have several potential therapeutic targets, such as VEGF, which is the most important signal mediating vascular growth, and several VEGF inhibitors are currently being tested in phase I or II trials for colon and lung cancer (60). Pirfenidone, an orally active synthetic anti-fibrotic, which not only reduces the risk of lung cancer in patients with idiopathic pulmonary fibrosis, but also inhibits tumor growth and distant metastasis of refractory breast and pancreatic cancer (64–66). Anti-fibrosis therapy holds great promise.

### Tumor-Associated Macrophages and Myeloid-Derived Suppressor Cells

Tumor-associated macrophages (TAMs), one of the most dominant immune cells in TME, can promote the growth and metastasis of tumor cells in many ways (67). In addition, TAMs in some settings stimulate anti-tumor immunity or kill tumor cells directly (68). Macrophages differentiate into typically activated macrophages (M1), induced by IFN-γ and/or lipopolysaccharide (LPS), which is important to host defense and anti-tumor immunity and activated macrophages (M2), induced by IL-4/IL-13, which play a critical role in fibrosis, promote wound healing, dampen inflammation and tumorigenesis (46, 69). TAMs play an M2 role to produce high levels of reactive oxygen free radicals, promote DNA damage and genomic instability, tumor infiltration and metastasis, participate in the digestion and reconstruction of extracellular matrix (ECM), inhibit anti-tumor immunity and so on (70). IFN-γ and celecoxib inhibits M2 differentiation, thus inhibiting tumor growth (71). Tumor cells release too much tumor-promoting and angiogenic cytokines/chemokines with TAMs and tumor-associated neutrophils (TANs), targeting these mediators and blocking immunosuppressive molecules expressed by tumor cells or tumor-infiltrating immune cells (9).

Myeloid-derived suppressor cells (MDSCs) are also one of the important components of TME (72). MDSCs are an effective inhibitor of innate and adaptive immunity, especially on T cells (73, 74). MDSCs can induce CD8^+^ T cell tolerance, this CD8^+^ T cell tolerance is one of the major mechanisms of tumor escape. The specific manifestation is that MDSCs induce the nitration of TCR/CD8 complex through the excessive production of reactive oxygen species (ROS) and peroxynitrite in the process of cell-cell direct contact, which leads to the inability of CD8^+^ T cells to bind to peptide-MHC (pMHC) and affect the ability to respond to non-specific stimulation (75). In addition, MDSCs increase the metabolism of L-arginine (L-Arg) by producing arginase I, which inhibits T cell-lymphocyte reaction and block T-cell activation by consuming cysteine (76, 77).

The stimulating factor 1 receptor (CSF1R) is significantly expressed in MDSCs and TAMs, which cause functionally reprogram the response of macrophage and enhance antigen presentation and anti-tumor T cell response. Meanwhile, T cell checkpoint molecules, including PDL1 and CTLA4, are upregulated by CSF1R blockade, thus inhibiting beneficial therapeutic effects, but the combination of PD1 and CTLA4 antagonists could enhance the efficacy of checkpoint-based immunotherapics (78). Other tumor-infiltrating cells of myeloid lineage such as TANs, releasing excessive amounts of pro-tumor and pro-angiogenic cytokines/chemokines. Targeting these mediators, blocking immunosuppressive molecules expressed by tumor cells or tumor-infiltrating immune cells, and promoting anti-tumor immune responses, which can effectively treat a variety of tumors (9). Thus, TAMs and MDSCs affect the growth and metastasis of tumor cells and the efficacy of tumor immunotherapy through a variety of channels.

### Cell Metabolism and Other Tumor-Infiltrating Cells

Cellular metabolism also has a critical important effect on the viability and function of both cancer cells and immune cells (79). Cancer cells up-regulate the absorption of nutrients and the production of waste metabolites, thus creating an immunosuppressive TME that allows it to escape and grow, and determines the fate of immune cells (80). Cell metabolism can be regulated using a combination of metabolic disruptors and immune checkpoint blockade (81). In addition to consuming key nutrients, tumors also produce large amounts of waste products such as lactic acid, arginine and tryptophan byproducts, and phosphoenolpyruvate, which can impair T-cell metabolism and function, then lead to a worse prognosis for patients (82). So cell metabolism becomes an attractive target for restoring anti-tumor immunity and developing anticancer therapies (81). Lactic acid is the primary cause of acidic PH and the inhibition of pH-dependent T cell function in the tumor micro-environment. Lactic acid generated by tumors and acidification of the microenvironment improves the expression of ARG1 in TAMs, which is characteristic of the M2-assisted phenotype (83). The inhibition of the production of lactic acid in cancer cells helps to recover active oxygen homeostasis of physiological mitochondrial and restore normal function of cells (84). Therefore, the recovery of the anticancer immune response can be achieved by targeting inhibition of the lactic acid production pathway (85).

To sum up, it is strongly demonstrated that TME plays a critical role in tumor immunotherapy. But solutions to these two problems is that overcome the inherent immunosuppressive tumor environment and stimulate a strong adaptive response (8).

## The Effect of Gut Microbiota and Its Metabolites on the Host Immune System Affects TME Shaping

**Figure 2:**
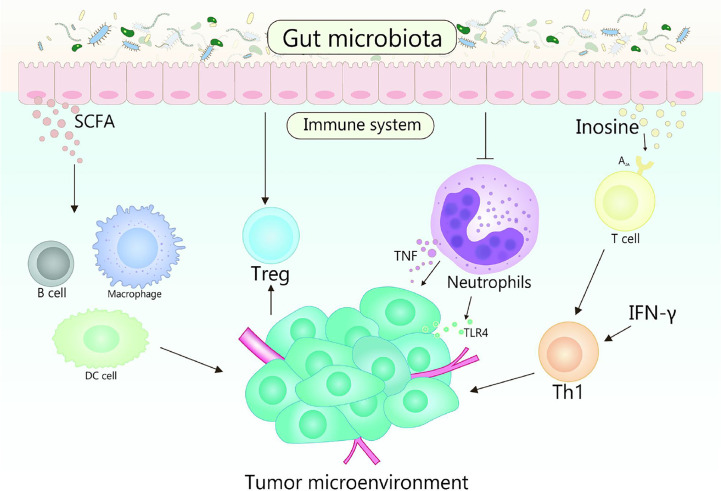
Gut microbiota and its metabolites act on the host immune system to influence the shaping of the TME. TLR4 signaling in tumor cells is able to recruit neutrophils, while TNF released by neutrophils is able to induce metastasis of tumor cells. Gut microbiota is beneficial to reduce the number of neutrophils, which plays a promoting role in the treatment of tumors. Gut microbiota metabolite inosine significantly promotes the differentiation of Th1 cells in the presence of exogenous interferon-γ by acting on the A2A receptor on T cells, while SCFA can regulate the production of cytokines, affect the class conversion of B cells, activate DC cells and macrophages, and affect the differentiation of memory T cells, which also plays an important role in cancer therapy.

Gut microbiota plays fundamental roles in the development in the function, maintenance and development of the host immune system (86, 87). In the early stage of life, gut microbiota shapes the immune system, and the changes of gut microbiota will affect many aspects of the immune system in the later stage of life (88). Otherwise, the diversity of gut microbiota is crucial for the establishment of immune regulation networks (89). Generally, multiple gut microbiota establishes a symbiotic relationship with the host immune system and promotes the host homeostasis, the perturbation of this relationship will result in chronic inflammatory and autoimmune immunopathology, thereby may causing or aggravating the formation and development of cancer (90). TME is the environment in which tumors grow, it can regulate tumor growth, promote tumor invasion and metastasis, mediate tumor immune escape, and promote or weaken the carcinogenic process (86, 91). The crosstalk between gut microbiomes and microbiome metabolites in TME is continuous and beneficial, that affects the TME by affecting host immunity and intestinal epithelium, and promotes or inhibits the development of tumor (**Figure 2**) (53, 92). For example, the efficacy of conventional chemotherapy and immunotherapy for pancreatic cancer are affected by the involvement of gut microbiota in the metabolism of TME (93). Based on the close interaction between host microbiota and immune response in TME, it is suggested that manipulating gut microbiota is a feasible strategy for anticancer therapy (94).

### Interaction Between Gut Microbiota and Microbiome Metabolites With Host Immunity Affects TME

Normal or pathological immune response will occur in the tumor treatment (95). Gut microbiota modulates the whole host immune, which impacts the distant preneoplastic lesions toward malignancy or regression. The interaction between gut microbiota and host immune enhances the possibility that the TME interacts with broader systemic microbial-immune networks, that reminds that the gut microbiota is emerging as an essential modulator of TME (96–100).

It is known that among immune cells, neutrophils and Treg as key cells in cancer development and growth (98, 101–103). Neutrophils can activate the interaction between cancer cells and endothelial cells in the primary TME, thus promoting tumor metastasis. In melanoma, neutrophils recruited by Toll-like receptor 4 (TLR4) signal can induce cancer cells to migrate to endothelial cells through tumor necrosis factor (TNF), resulting in enhancing cancer metastasis (104). Meanwhile, the adhesion of cancer cells is mediated by neutrophil Mac-1/ICAM-1, thus affecting its metastasis (1). Moreover, cytokines, chemokines, growth factors, and serine proteases of neutrophils shape microenvironment that contribute to the tumor growth (102). Cytokines released by tumor and TME send out an emergency signal to stimulate a large number of neutrophils to enter the blood circulation and accelerate the metastasis of cancer cells (105). However, the number of neutrophils in circulation is reduced by abundant gut microbiota. One research found the mice applying with *L.reuteri* showed a better capacity of wound healing, which is achieved by reducing the number of neutrophils in circulation through the increase of Foxp3 and Tregs (106). Another research pointed out the same result that the cachexia mice treated with *L.reuteri* showed decreased systemic inflammation and better tumor inhibition, which also associated with the reduction of neutrophils in the blood (107). And, the neutrophil homeostasis will be affected by microbiota through enterocyte CXCL5-mediated signaling and IL-17 (108).

Treg is essential for maintaining the homeostasis of the immune system and the balance of beneficial inflammatory response during infection (109). Treg regulates the host immune response which gathers near TME, suppresses the anti-tumor inflammatory response and counteracts antigen-specific effector T-cell responses, consequently (109–111). The differentiation and proliferation of Treg, and the secretion and recruitment of immunosuppressive factors will be activated by TME, then contribute to the immunosuppression of tumor tissue (112). Based on the data of animal models, it has been found that the Treg induced by some specific gut microbiota could change the TME, which is beneficial to relieve the induction of cancer. For example, CD4^+^ CD25^+^ Treg cells inhibit the occurrence of colon cancer by inhibiting the development of *H. hepaticus*-induced inflammation and dysplasia (113), the effectiveness of Treg cells will be enhanced by the infection from gut pathogens, thereby inhibiting the occurrence of breast cancer (114). Gut microbiota response mediated by IL22^+^ innate lymphoid cells, Th17 cells and Treg cells occurred in mice lacking adaptive immunity, indicating that gut microbiota can promote the innate immunity (115). The mechanism of tumor reduction driven by microbiota may induce the anticancer immune response (102, 116).

Otherwise, the intestinal epithelial barrier, a physical barrier, that are extremely essential in maintaining the balance of the intestinal environment (117). On the one hand, the common gut microbiota can enhance immunoglobulin A production in the intestinal tract by regulating the response of B cells to maintain an intact epithelial barrier (118, 119), which also is a key figure in the development of the immune system (92, 120). Once this barrier is broken, the main inflammation-activating transcription factor NF-κB will be activated (121). The activation of NF-κB in ovarian cancer cells responds to inflammatory chemokines and cytokines in the TME, which helps to create an immune escape environment and attract infiltrating immune cells with tumor-promoting phenotypes (122). The origin of tumor-promoting inflammation is quite clear in gastrointestinal cancer, most of which can be attributed to the destruction of epithelial barrier integrity (123). On the other hand, intestinal epithelial cells can activate the NOTCH1 signal and lead to a high penetrating transfer of colorectal cancer (124).

Thus, the interaction between neutrophils and TME accelerates the progression of tumors, and the dynamic balance of neutrophils is affected by gut microbiota. Treg gathers near TME with an immunosuppressive effect, which is induced by specific gut microbiota to changes TME, thereby alleviate cancer. Maintaining the integrity of the intestinal epithelium can be maintained by gut microbiota, which is conducive to reduce e the incidence of cancer.

### Interaction Between Gut Microbiota Metabolites and Host Immunity Affects TME

Gut microbiota metabolites enter host cells and mutually interact thereby affecting the immune response and disease risk, promote a variety of tumor inhibitory and immunomodulatory effects, and inhibit inflammation by maintaining the integrity of epithelial barrier and intestinal tract (9, 125). Accumulating evidence suggests that gut microbiota metabolites and products of their metabolic activities influence important metabolic pathways of the host related to food intake, adiposity, lipid and energy homeostasis (114, 126–131).

Some fatty acids and cholic acids are related to inflammation. SCFAs contribute to maintaining intestinal homeostasis and regulating intestines’ barrier function (132, 133). It acts on G protein-coupled receptors (GPCRs) to inhibit the metastasis of breast cancer (134). Butyrate, an SCFAs, produced by *Faecalibacterium prausnitzii*, which has the ability to suppress angiogenesis and reduce the expression of pro-angiogenic factors, so increasing the concentration of butyrate can play a role in protecting and preventing cancer (135–137). In addition, Deoxycholic acid (DCA) and petrocholic acid (LCA) potentially cause DNA damage by enhancing the production of ROS (138). DNA damage causes cell senescence, chronic inflammation, and tumorigenesis (139). Recent studies have suggested that the metabolite inosine was produced by intestinal bacteria *B. pseudolongum*, which significantly promoted Th1 cell differentiation in the presence of exogenous IFN-γ and enhanced the therapeutic response of ICB therapy including anti–CTLA-4 and anti–PD-L1, by acting on adenosine A_2A_ receptor on T cells (12). Understanding how the metabolites and sub-metabolites of gut microbiota affect immune cell subsets and their actions to reshape TME may be the direction of future research.

Specific gut microbiota interacts with immune cells to promote tumor clearance, slow metastasis of cancer cells and inhibit chronic inflammation, thus mitigating against cancer. The gut microbiota metabolites, such as SCFAs and inosine, directly or indirectly interact with TME to reshape TME, and affect the cancer process. However, a study has found that some gut microbiota (such as *Bacteroides* and *Ruminococcaceae*) can participate in the occurrence of HCC by aggravating hepatocyte inflammation, accumulating toxic compounds and leading to liver steatosis (23). Therefore, a comprehensive understanding of the interaction mechanism between gut microbiota and its metabolites with the host immune system in reshaping and regulating TME through gut microbiota is profound for cancer immunotherapy.

## The Effect of Gut Microbiota in Cancer Immunotherapy

**Figure 3:**
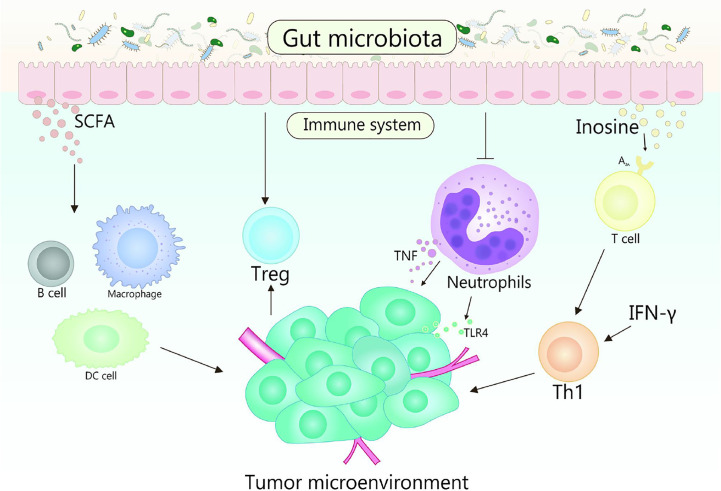
Role of gut microbiota in cancer immunotherapy. Gut microbes can stimulate the body to produce CD47 antibodies by activating STING signaling, thereby promoting immunotherapy. It is reported that the cross-priming of antigen-specific T cells of tumor-resident DCs can be enhanced by anti-CD47 therapy. In addition, type I IFN plays an important role in enhancing the adaptive immune response to anti-CD47 antibody therapy in tumor-resident DCs. Accumulation of *Bifidobacteria* in the TME can significantly improve the antitumor efficacy of anti-CD47 immunotherapy, which is dependent on STING signaling and type I IFN within DCs. *Bifidobacteria* may affect activating DC cells, thereby improving the activity of tumor-specific CD8^+^ T cells. The key role of B. fragilis is to restore the anti–CTLA-4 treatment response associated with Th1 immune responses in tumor-draining lymph nodes.

Immunotherapy realizes the result of eliminating tumors by suppressing negative immune regulatory factors, activating the immune system and enhancing the recognition, thereby killing of immune cells to tumors (140). With further research, it has been verified that the gut microbiota could regulate the immune response, thus affect the effectiveness of cancer immunotherapy (120, 141). Various mechanisms mediated by gut microbiota affect the therapeutic response and toxicity of immune checkpoint inhibitors (ICIs), chemotherapy, and stem cell transplant (**Figure 3**) (142). In fact, some studies indicate that there is a strong correlation between gut microbiota and immune checkpoint response (143–145). Gut microorganisms play a crucial role in cancer treatment by eliminating anticancer effects and mediating toxicity.

### Anti-CD47 Therapy

Tumor immunotherapy identified and kills tumor cells through the host immune system, and more and more attention has been paid to the research in this field (146–148). CD47, as a signal molecule to help tumor cells escape, conveys the “don’t eat me” signal to immune cells and produces a good effect of tumor immunotherapy by blocking the CD47 (149, 150). CD47 is a kind of transmembrane protein that has interactions with signal regulatory protein (SIRP) expressed on macrophages and DCs (151). It is a hint of a poor prognosis when CD47 can highly express in malignant tumors, for example, leukemia, myeloma, ovarian tumor, and so on (152, 153).

Anti-CD47 antibody therapy makes not only cancer cells swallowed by macrophages but also anti-tumor cytotoxic T cell immune response initiated (154). Researches have shown that CD8^+^ T cells are essential for anti-CD47-mediated tumor regression, and tumor-resident DCs can enhance anti-CD47 by cross-stimulation of antigen-specific T cells (150). In addition, DC-specific type I IFN plays a critical role in enhancing the adaptive immune response to anti-CD47 antibody therapy (149, 150). A research pointed out that the main anti-tumor effect of anti-CD47 monoclonal antibodies is attributed to the activation of host cGAS-STING pathway mediated by mitochondrial DNA in DC (150).

Anaerobes play a dominant role in the gastrointestinal tract, among common anaerobes, *Bifidobacterium* is a functional gut microbiota, which is widely used in the treatment of inflammatory gastrointestinal diseases such as ulcerative colitis (155, 156). TME in a low oxygen state creates a good growth environment for anaerobic bacteria (157). A study indicated that the accumulation of *Bifidobacterium* in TME can significantly improve the anti-tumor effect of anti-CD47 immunotherapy, which depends on the STING signal and the type I IFN within DC (158). CD47 is widely expressed in a variety of solid human tumors. At present, the related mechanism of CD47 has been extensive and in-depth research. However, there are few studies on the influence of gut microbiota on anti-CD47 immunotherapy, which has a great prospect.

### Interaction Between Gut Microbiota and ICIs

Immune checkpoints are a type of membrane‐bound molecules, which can impede uncontrolled T‐cell response after initial stimulation. This mechanism can be used for cancer cells to escape immune surveillance. ICIs, however, can reactivate the inefficient T cells and recover the response to tumor antigens (159). Based on the current research background, CTLA-4, PD-1, and PD-L1 are the most in-depth target of immune checkpoint therapy. CTLA-4, PD-1, or PD-L1 have shown strong antitumor activity in the experimental animal models and the long-lasting clinical efficacy in cancer patients, such as melanoma, renal cell cancer, and lung cancer (159). Clinical studies and preclinical trials have shown that the efficacy of ICIs is affected by gut microbiota, which explains the large individual differences in patients’ responses to ICIs (160, 161). At present, a variety of inhibitions about them have been invented and used or used or tested in the clinic (**Table 1**). Obviously, the response of ICIs is closely decided by the diversity and composition of gut microbiota (160, 162).

**Table 1:** Modulatory function of gut microbiome in ICIs therapy.

Bacteria	Model	Modulatory function of gut microbiome in ICIs therapy	References
*Bifidobacterium adolescentis*	Mouse	(1) Stimulating DCs directly, inducing DCs maturation and cytokine secretion (2) Anti-tumor function and synergistic effect with PD-1 blockade	(160)
*Bacteroides fragilis*	Mouse	(1) Immunostimulation induced by CTLA-4 blockade (2) Promoting the maturation of DCs in tumor cells and inducing Th1 cell activation	(161)
*Bacteroides thetaiotaomicron*	Mouse	(1) Anti-tumor effect of CTLA-4 blockade (2) Promoting Th1 immune response	(161)
*Blautia obeum*	Human	Related to impaired efficacy of PD-1 blockers	(103)
*Collinsella aerofaciens*	Human	(1) Results in decreased peripheral blood Treg (2) Associated with enhanced efficacy of PD-1 blockade	(103)
*Enterococcus faecium*	Human	(1) Results in decreased peripheral blood Treg (2) Associated with enhanced efficacy of PD-1 blockade	(103)
*Klebsiella pneumonia*	Human	(1) Associated with enhanced efficacy of PD-1 blockade	(103)
*Parabacteroides merdae*	Human	(1) Results in decreased peripheral blood Treg (2) Associated with enhanced efficacy of PD-1 blockade	(103)
*Roseburia intestinalis*	Human	Related to impaired efficacy of PD-1 blockers	(103)
*Veillonella parvula*	Human	Associated with enhanced efficacy of PD-1 blockade	(103)
*Ruminococcaceae*	Human/mouse	(1) Associated with enhanced efficacy of PD-1 blockade (2) Increasing effector T cell levels in peripheral blood and TILs	(11)
*Enterococcus hirae*	Human/mouse	(1) Synergistic effect with PD-1 blockers in combination with Ackerman mucilaginosa (2) Improving traditional Chinese medicine combined with Ackerella Sinensis	(12)
*Alistipes indistinctus*	Human/mouse	Restoring the anti-tumor efficacy of PD-1 blockers	(12)

PD-1 and PD-L1 are members of the immune checkpoint proteins relating to the suppression of the immune system and delivering inhibitory signals to T cells (163). Cancer immunotherapy targeting PD-L1 and PD-1 has been widely carried out, and gut microbiota has been proposed to affect its efficacy and toxicity. Maston et al. analysis of 38 fecal samples from patients with metastatic melanoma who received anti-PD1 therapy, it is found that *Bifidobacterium longum*, *Enterococcus faecalis*, and *Collinsella aerofaciens* contribute to a better prognosis (145). Researchers transferred the fecal material from Jackson Laboratory (JAX) or Taconic Farms (TAC) from one mouse to another by oral gavage before tumor implantation and pointed out that the DCs may be activated by the increasing abundance of *Bifidobacterium longum*, thus improve tumor‐specific CD8^+^ T cells activity (160). With researches obtain more attention to identifying the specific bacteria genres that play a critical role in human immunity by clinical experiments. Gopalakrishnan et al. found the melanoma patient respond to anti-PD1 treatment has higher microbial diversity, including the abundance of *Ruminococcaceae*, *Clostridiales*, and *Faecalibacterium*, patients with more *Faecalibacterium* has a significantly prolonged progression-free survival with a higher level of effector T cells and a stabilized cytokine response to PD-1 blockade, simultaneously, systemic and anti-tumor immunity are also enhanced (144). Another research also achieved the same opinions, they found the *Alistipes putredinis*, *Bifidobacterium longum*, and *Prevotella copri* were enriched in responsive patients with advanced non-small-cell lung carcinoma who were being treated with PD-1 blockade therapies, expectedly, a greater frequency of memory CD8^+^ T cell and NK cell subgroups was observed in the periphery blood of responding patients (164). The abundance of the gut microbial flora acting as immune adjuvants in the immunotherapy of PD-1 and the T cell response may deeply connect with the PD-1/PD-L1 immunotherapy, and relevant researches have shown that the patients with endogenous T cell response in TME are more effective in immunotherapy (165, 166). The hot tumor with a large number of T cell infiltration, which has the highest response rate to tumor immunotherapy (167). Targeted inhibition of Vps34, can transform “cold tumor” into “hot tumor”, thus enhancing the efficacy of PD-L1/PD-1 blocking therapy (168).

CTLA-4, also known as CD152, is constitutively expressed in Tregs and acts as an immune checkpoint that decreases immune responses (169). Vetizou et al. found that a key role of *Bacteroides thetaiotaomicron* or *Bacteroides fragilis* restores response to the anti–CTLA-4 therapy associated with T-helper 1 immune responses in tumor-draining lymph nodes and maturation of intratumoral DCs, activation of effector CD4+ T cells and TILs elicited by CTLA-4 blockade was considerably dampened in germ-free or antibiotics mice, otherwise, the intestinal reconstitution of antibiotic-treated mice with *Bacteroides* and *Burkholderia* genres could restore the CTLA-4 blockade-mediated anticancer responses (161). The abundance of *Bacteroides* in patients with new immune-mediated colitis treated with anti–CTLA-4 was significantly lower than that in patients without colitis treated with ipilimumab. Meanwhile, the response of mice to anti-CTLA4 antibody could be restored and the degree of immune-mediated colitis could be significantly reduced by taken orally administration of *Bacillus fragilis* and *Bacillus cepacia* (170). However, a single dose of *Bacillus fragilis* or *B.thaiotaomicron* could not receive the same effect (161, 171). Vancomycin enhances the blocking effect of CTLA-4 by increasing the proportion of Gram-negative *Burkholderia* and *Bacteroides* in the intestines (161). It may prove that *Bacillus* can be used to regulate the efficacy of anti–CTLA-4 therapy.

Another research suggested that the special gut microbiota contributes to both CTLA4 and anti–PD-L1 immunotherapy. Three bacteria from the intestinal tract, *Bifidobacterium pseudolongum*, *Lactobacillus johnsonii*, and *Olsenella* from the intestinal tract significantly enhanced the efficacy of anti-CTLA4 and anti–PD-L1 immunotherapy in four different cancer mice when they were introduced into aseptic mice with ICIs, and the *Bifidobacterium pseudolongum* in the intestine contributes to regulating and enhancing the immunotherapeutic response by producing inosine (12). Based on these reports suggest that the commensal microbiome may have a mechanistic impact on antitumor immunity in cancer patients, and a growing number of studies have also emphasized that the gut microbiota could modulate response to cancer immunotherapy. Consequently, the further research about the effect and the potential mechanism of gut microbiota in ICIs is profound for cancer treatment. Innovative treatments were used to study and not widely applicate in patients, so it is necessary that further works must unlock the mystery of microbial modulation in various anticancer immunotherapies.

### Gut Microbiota Affect the Efficacy of Cancer Immunotherapy

Abundant gut microbiota plays a regulatory role in tumor therapy, which has a critical role in regulating the efficacy and toxicity of cancer immunotherapy (172). The effect of gut microbiota on the efficacy and interaction of ICIS has been verified in melanoma, non-small cell lung cancer, urethral epithelial carcinoma, and renal cell carcinoma (144, 145, 173). Lukas F Mager et al. found that *Bifidobacterium pseudolongum*, *Lactobacillus johnsonii*, and *Olsenella* enhanced efficacy quadrupled of ICIs in four mouse models of cancer (12). It is suggested that selective regulation of gut microbial population may help to overcome the resistance to ICIs (159, 167).

Among radiotherapy, ionizing radiation therapy (RTX) is an effective method for tumors treatment, but severe oral mucositis and bowel disease caused by RTX may limit the completion of treatment, fortunately, the probiotics such as Lactobacillus casei, Rhamnose and Bifidobacterium have been shown to reduce radiotherapy-associated diarrhoea in mouse models by inhibiting the expression of TNF, IL1b, and IL6mRNA (174–176). It is reported that complete response of tumor cells with local temperature causing by infrared radiation of a specific wavelength, and the formation of the tumor specific thrombus can achieve effective photothermal immunotherapy of cancer through the action of attenuated Salmonella in an innovative photothermal therapy (177). On the other hand, gut microbiota may reduce the side effects of a variety of chemotherapy. In chemotherapy, cyclophosphamide (CTX), one of the most commonly used chemotherapeutic drugs in treating lymphomas and solid tumors, inducing immunogenic cancer cell death and immunomodulatory effects (178). Orally administrated with Enterococcus hirae cause a restoration of CTX anti-tumor efficacy by inducing differentiation of TH17 and pathogenic TH17 cells, promoting tumor-specific Th1 and CTL activity (179). Abiraterone acetate is both an inhibitor of androgen biosynthesis and a highly effective drug of prostate cancer, which reduces the harmful microorganisms and promotes the growth of anticancer microorganisms through metabolizing the gut microbiota (180). Otherwise, Pushalkar et al. suggested that bacterial ablation can reshape TME in the orthotopic mouse model of pancreatic cancer (PDAC), induce activation of T cell and increase the sensitivity of immunotherapy (181).

Excepting the related advantages, the correlation between the composition of intestinal microbial community and the degree of TNF shows that some *Lactobacillus strains*, such as fermented *Lactobacillus*, are considered as weakening the response to immunotherapy (182). The composition of gut microbiota is related to the different development of graft-versus-host disease (GVHD). It has high morbidity and mortality, when the cross reaction occurs between the donor cells (usually T cells) the graft and the patient’s major histocompatibility. On the contrary, it was found that increased bacterial diversity and increased amounts of *Blautia* to be related to reduce GVHD mortality and improve survival (183).

At present, research has moved away from the approach based on association toward mechanism as the advances in sequencing technology and the development of powerful computing tools. Elucidating the relationship between the gut microbiome and cancer as well as the potential mechanism has become the priorities of further research, which also the main method for cancer immunotherapy.

## Conclusion and Future Perspective

The gut microbiota activates the host immune system further and has an anti-cancer effect, and more superiority than the traditional way of treating cancer. In addition, the interaction between gut microbiota and cancer ICIs play an antitumor immune therapy, this way of targeted therapy in cancer immunotherapy is getting more and more recognition. Targeting and manipulating cells and factors in TME during cancer therapy, which contribute to control malignancies and obtain positive health outcomes (132). An in-depth understanding of TME, its role and related molecules will provide important insights into the biological behavior of different tumor types. Molecules and tumorigenic processes in TME are considered as the key targets of the new therapy strategy of cancer (86, 184). The refining of molecular cells and immune regulation of therapeutic targets is increasing in the TME, and the clinical application also is growing more and more widely, for example, that resistance to PD-1/PD-L1 plays multiple roles in tumor immunotherapy, however, the test of limited activity PD-1 of resisting tumor types may have a good therapeutic effect in the strategy of reshaping the tumor inert environment in the future. Namely the possibility of immunotoxicity and immunotherapy to enhance antitumor immunity, in other words, use reasonable and selective combined immunotherapy in a limited TME to reactivate the anti-tumor immune response (185).

In the previous introduction, we have known that gut microbiota is essential in maintaining the host balance, promoting physiological responses including the protection of pathogen, host metabolism, host immunity response, and so on. More and more scientific evidence showed that broken the delicate balance of gut microbiota can lead to the occurrence of cancer and other diseases, which indicates that modulating strategy is very important. In fact, gut microbiota has great inter-individual heterogeneity due to the impact of host, including the age, living environment, genetic factor, and diet habit. Among them, diet habit and host age are the main determinants of gut microbiota according to the biological relationship between gut microbiota and host in nutrient digestion (186). It is reported that the diversity of gut microbiota and its metabolites were changed by diet habit (187). The overall richness of gut microbiota decreases with age, and some microbial taxa related to unhealthy aging appear, which leads to the malnutrition of gut microbiota, then finally affect the host’s innate immune response (188). Fecal microbiota transplant (FMT), live biotherapeutics, diet habits, and prebiotics are the main strategies to regulate gut microbiota, making it more healthy (189). For example, patients with colon cancer have obvious characteristics and diversity of gut microbiota in tumor tissue and nearby mucosa. After taking probiotics, the abundance of *butyrate-producing bacteria* in tumors, non-tumor mucosa, and fecal flora increased in these patients, that helps maintain the intact intestinal barrier to avoid the activation of inflammation-related factors in TME (190, 191). The report pointed out that non-responders lack beneficial bacteria which are critical to the anti-tumor effect of immunotherapy, however, the effect of them on ICIS can be restored by transplanting these bacteria from responders to non-responders by some means, such as FMT, probiotic therapy, and so on (192).

In addition, determining the composition of individual gut microbiota is also a way to solve the huge heterogeneity among individuals in the gut microbiota. Searching for microbial signals to determine the degree of response to cancer treatment may help determine gut microbiota composition associated with specific treatment categories or overlapping signals, which is suitable for a wide range of immune therapy. But the microbe signal is a continuous work, the differences of sequencing technology and patient cohort were all factors that affect microbial expression (193). The composition of gut microbiota havs undergone a similar change during cancer progression and treatment, and this change causes more additional challenges. Cancer cells grow and evolve under the therapy of selective pressure. Molecular evolution of tumors may still occur when gut microbiota is manipulated to maximize immunotherapeutic efficacy. Therefore, future research may be able to use DNA sequencing, metabolomics technologies and high-dimensional data, as well as to give them intervention, the interdependence of individual host-intestinal microflora can provide more effective treatment and greatly promote the development of microbial alliance to treat specific disease (194). More challenges need to be overcome by combination with basic experimental and clinical research. Therefore, we look forward to getting more precise therapies targeted cancer coming from gut microbiota and the TME in the future. It is also suggested that further studies should focus on the precise targets and mechanisms of action in this field.

## Author Contributions

LL and RH conceived and designed the review. QQ, YM, YL, ZC, JL, XL, and LL wrote the manuscript. LL, YH, and HL reviewed the paper and provided comments. All authors contributed to the article and approved the submitted version.

## Funding

This project was supported by the Ph.D. Start-up Fund of Guangdong Medical University (B2019016); Administration of Traditional Chinese Medicine of Guangdong Province (20201180); Science and Technology Special Project of Zhanjiang (2019A01009); Natural Science Foundation of Guangdong Province (2016B030309002); Basic and Applied Basic Research Program of Guangdong Province (2019A1515110201); GDNRC[2020]038; Educational Commission of Guangdong Province (4SG20138G); and Fund of Southern Marine Science and Engineering Guangdong Laboratory (Zhanjiang) (ZJW-2019-007).

## Conflict of Interest

The authors declare that the research was conducted in the absence of any commercial or financial relationships that could be construed as a potential conflict of interest.
